# Trends and disparities in intrahepatic cholangiocarcinoma mortality in the United States, 1999–2024: a CDC WONDER analysis

**DOI:** 10.3389/fonc.2026.1910061

**Published:** 2026-08-14

**Authors:** Jianguo Chen, Guangwei Yang, Xiaodong Sun

**Affiliations:** Department of Hepatobiliary and Pancreatic Surgery, General Surgery Center, The First Hospital of Jilin University, Changchun, China

**Keywords:** age-adjusted mortality rate, CDC WONDER, health disparities, intrahepatic cholangiocarcinoma, joinpoint regression, mortality

## Abstract

**Background:**

Contemporary national estimates of intrahepatic cholangiocarcinoma (ICC) mortality extending through 2024 are limited. We examined long-term temporal trends and demographic and geographic disparities in ICC mortality among adults in the United States.

**Methods:**

We conducted a serial cross-sectional analysis of the Centers for Disease Control and Prevention Wide-ranging Online Data for Epidemiologic Research (CDC WONDER) Underlying Cause of Death databases. Deaths among US adults aged 25 years or older from 1999 through 2024 were identified using International Classification of Diseases, 10th Revision code C22.1 as the underlying cause of death. Mortality was examined by year, sex, age, race and ethnicity, US Census region, and state from 1999 through 2024; urban–rural analyses were restricted to 1999–2020 because later data were unavailable in the extracted database. Crude mortality rates and age-adjusted mortality rates (AAMRs) per 100,000 population were calculated. Joinpoint regression was used to estimate annual percent changes (APCs) and average annual percent changes (AAPCs) with 95% confidence intervals (CIs).

**Results:**

From 1999 to 2024, 143,345 ICC deaths were recorded among adults aged 25 years or older. Annual deaths increased from 2,552 to 9,752 (282.13%), and the AAMR rose from 1.44 to 3.35 per 100,000 (AAPC, 3.38%; 95% CI, 3.24%–3.52%). In 2024, the AAMR was higher in men than women (3.70 vs 3.11), although the increase was faster in women (AAPC, 3.56% vs 3.15%). Adults aged 85 years or older had the highest crude mortality rate (16.89), whereas those aged 35–44 years had the fastest increase (AAPC, 4.13%). Hispanic individuals had the highest AAMR (3.47), while non-Hispanic Black individuals had the largest AAPC (4.03%). The Midwest had both the highest AAMR (3.68) and fastest increase (AAPC, 3.62%). From 1999 to 2020, metropolitan areas had slightly higher AAMRs and faster increases than nonmetropolitan areas.

**Conclusions:**

Mortality coded to ICC increased substantially in the United States from 1999 through 2024, with marked heterogeneity by sex, age, race and ethnicity, and geography. These descriptive findings distinguish populations with high absolute mortality from those with rapid relative increases, but they do not establish the causes of the observed trends.

## 1 Introduction

Intrahepatic cholangiocarcinoma (ICC) is an epithelial malignancy arising from the intrahepatic bile ducts proximal to the second-order branches. It accounts for approximately 10%–15% of primary hepatic malignancies and is the second most common primary liver cancer after hepatocellular carcinoma. Because early-stage ICC is frequently asymptomatic or presents with nonspecific symptoms, many patients are diagnosed with locally advanced or metastatic disease and are not candidates for curative resection. Despite advances in hepatobiliary surgery, systemic therapy, molecularly targeted therapy, and immunotherapy, ICC continues to confer a poor prognosis and represents an increasingly important clinical and public health challenge (1, 2).

Population-based studies using cancer registries, vital statistics systems, and the CDC WONDER database have reported increasing incidence and mortality from primary liver cancer, ICC, and other intrahepatic bile duct malignancies in the United States and elsewhere over recent decades (2–11). Analyses covering 1999–2020 consistently showed a growing ICC mortality burden and substantial variation by age, sex, race and ethnicity, and geographic region (2, 12–14). These patterns may reflect a combination of changes in population structure, the prevalence of established and emerging risk factors, diagnostic ascertainment, disease coding, and access to timely oncologic care.

The risk profile of ICC is heterogeneous and includes chronic biliary inflammation, primary sclerosing cholangitis, cirrhosis, metabolic disorders, chronic hepatitis B or C virus infection, and selected environmental exposures (1, 8, 15, 16). Persistent disparities in cancer and liver disease mortality across racial and ethnic groups, geographic regions, and urban–rural settings further suggest that social determinants of health and differences in healthcare access may shape the observed burden (17–20). In parallel, the increasing burden of early-onset gastrointestinal malignancies raises concern that the epidemiology of ICC among younger adults may also be changing (21).

Most prior national analyses of ICC mortality in the United States ended in 2020 and therefore did not capture mortality through 2024. The period after 2020 may have been accompanied by changes in healthcare utilization, chronic liver disease management, diagnostic pathways, and access to cancer care. We therefore used the most recent national mortality data available from CDC WONDER to characterize ICC mortality trends among US adults aged 25 years or older from 1999 through 2024. We also examined variation by sex, age, race and ethnicity, Census region, state, and urban–rural status to identify populations and locations with high absolute mortality or rapidly increasing rates.

## 2 Methods

### 2.1 Study design and data source

This serial cross-sectional study used national death certificate data from the CDC WONDER Underlying Cause of Death databases. To construct a continuous series from 1999 through 2024, we extracted aggregate data from the Underlying Cause of Death, 1999–2020 database and the Underlying Cause of Death, 2018–2024, Single Race database. The 1999–2020 database uses bridged-race categories, whereas the 2018–2024 database uses single-race categories. Data for 1999–2020 were obtained from the former and data for 2021–2024 from the latter; overlapping observations from 2018–2020 in the Single Race database were excluded to prevent duplicate counting. For race and ethnicity analyses, categories were harmonized to the four groups used throughout the study: Hispanic, non-Hispanic White, non-Hispanic Black, and non-Hispanic other race. Estimates from overlapping years were not pooled or used to calibrate the two databases. Because bridged-race and single-race classifications are not fully equivalent, longitudinal race and ethnicity comparisons spanning 2020–2021 were interpreted cautiously.

CDC WONDER provides publicly accessible, deidentified aggregate data and does not permit identification of individual decedents. Accordingly, institutional review board approval and informed consent were not required. The study was reported in accordance with the Strengthening the Reporting of Observational Studies in Epidemiology (STROBE) guideline.

### 2.2 Study population and case definition

We included deaths among US residents aged 25 years or older between 1999 and 2024 for which malignant neoplasm of the intrahepatic bile duct (International Classification of Diseases, 10th Revision [ICD-10] code C22.1) was listed as the underlying cause of death. Code C22.1 served as an operational proxy for ICC in death-certificate data. It does not confirm histology, tumor subtype, diagnostic workup, or exact anatomic origin and may include miscoded perihilar or extrahepatic cholangiocarcinoma, other primary liver or biliary tract cancers, or metastatic adenocarcinoma. Restriction to adults aged 25 years or older reduced the influence of sparse event counts and unstable estimates in younger age groups.

### 2.3 Stratification variables

Mortality was stratified by calendar year, sex, age group, race and ethnicity, US Census region, state, and urban–rural status. Age groups were 25–34, 35–44, 45–54, 55–64, 65–74, 75–84, and 85 years or older. Race and ethnicity were categorized as Hispanic, non-Hispanic White, non-Hispanic Black, and non-Hispanic other race. Census regions were Northeast, Midwest, South, and West. Urban–rural status was based on the National Center for Health Statistics Urban–Rural Classification Scheme and collapsed into metropolitan and nonmetropolitan categories. Because urban–rural data were not available for the full study period in the selected database structure, analyses by urban–rural status were restricted to 1999–2020.

### 2.4 Outcomes

The primary outcomes were the number of deaths, crude mortality rate, and age-adjusted mortality rate (AAMR). Rates were expressed per 100,000 population. AAMRs were calculated by direct standardization to the 2000 US standard population. Overall analyses and analyses by sex, race and ethnicity, Census region, state, and urban–rural status used AAMRs with 95% CIs. Age-stratified analyses used age-specific crude mortality rates because each stratum represented a defined age group.

### 2.5 Statistical analysis

We summarized deaths and mortality rates with 95% CIs for the first and last years of each analysis. The relative change in the number of deaths was calculated as follows: ([deaths in the final year − deaths in the initial year]/deaths in the initial year) × 100%. For overall, sex-, age-, race and ethnicity-, region-, and state-specific analyses, the initial and final years were 1999 and 2024, respectively. For urban–rural analyses, the corresponding years were 1999 and 2020.

Joinpoint regression was formally performed using the Joinpoint Regression Program, version 5.2.0.1 (National Cancer Institute). Weighted log-linear models used calendar year as the independent variable and the annual AAMR or age-specific crude mortality rate as the dependent variable. The standard error of each annual rate was incorporated to account for differences in precision across years. The maximum number of joinpoints was set to 4, and the optimal model was selected using permutation tests. The models generated segment-specific APCs, full-period AAPCs, and corresponding 95% CIs. A trend was considered statistically significant when the 95% CI for the AAPC excluded zero. All statistical tests were two-sided, and P <.05 was considered statistically significant.

CDC WONDER suppresses counts and rates when the number of deaths is small. Suppressed or unavailable annual counts, rates, and standard errors were neither imputed nor replaced with zero. Years with unavailable rate estimates or standard errors were excluded from trend modeling; when the remaining series did not support a reliable model, the APC or AAPC was reported as not estimable. All subgroup and state-level comparisons were exploratory, and no adjustment was made for multiple comparisons. Interpretation emphasized effect magnitude, consistency, and 95% CI precision.

## 3 Results

### 3.1 Overall mortality trends

**Figure 1:**
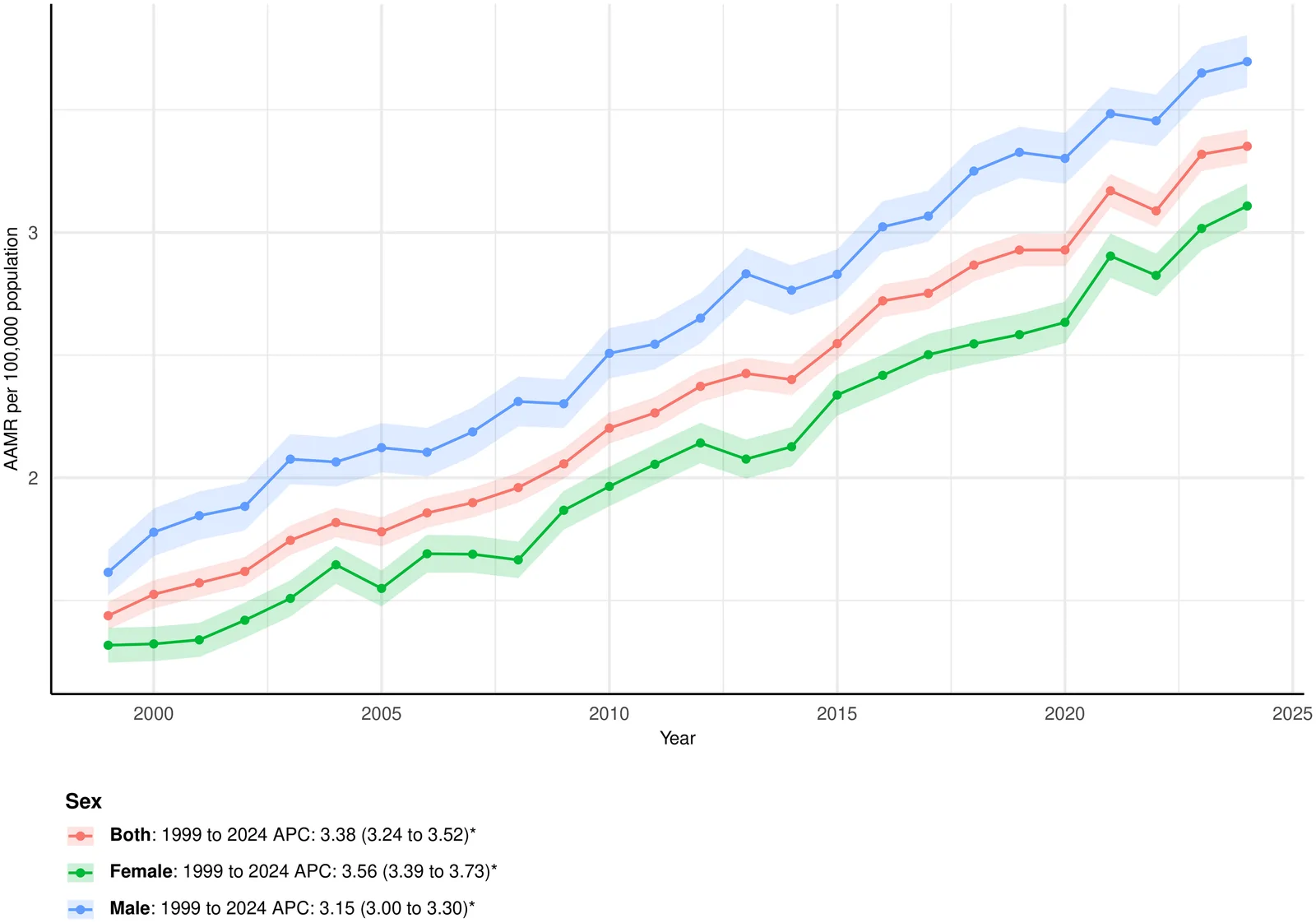
Trends in age-adjusted mortality rates for intrahepatic cholangiocarcinoma overall and by sex in the United States, 1999–2024. The analysis included decedents aged 25 years or older whose underlying cause of death was intrahepatic bile duct cancer (ICD-10 code C22.1). AAMRs were standardized to the 2000 US standard population and are reported per 100,000 population. Shaded areas represent 95% CIs. Asterisks indicate statistically significant APCs. AAMR, age-adjusted mortality rate; APC, annual percent change.

From 1999 through 2024, 143,345 deaths with ICC as the underlying cause were recorded among US adults aged 25 years or older. Annual deaths increased from 2,552 in 1999 to 9,752 in 2024, representing a 282.13% increase. Over the same period, the AAMR increased from 1.44 per 100,000 population (95% CI, 1.38–1.49) to 3.35 per 100,000 (95% CI, 3.28–3.42). The overall AAPC was 3.38% (95% CI, 3.24%–3.52%), indicating a statistically significant increase (**Table 1**; **Figure 1**).

**Table 1:** Deaths, AAMRs, and temporal trends of intrahepatic cholangiocarcinoma deaths overall and by sex, census region, and race/ethnicity in the United States, 1999–2024.

Characteristic	Deaths			AAMR		
	1999	2024	Percent change (%)	1999 (95% CI)	2024 (95% CI)	AAPC (95% CI)
Total	2552	9752	282.13	1.44 (1.38 to 1.49)	3.35 (3.28 to 3.42)	3.38 (3.24 to 3.52)*
Sex						
Female	1344	4840	260.12	1.32 (1.25 to 1.39)	3.11 (3.02 to 3.20)	3.56 (3.39 to 3.73)*
Male	1208	4912	306.62	1.61 (1.52 to 1.71)	3.70 (3.59 to 3.80)	3.15 (3.00 to 3.30)*
Census Region						
Northeast	551	1788	224.50	1.48 (1.36 to 1.60)	3.38 (3.22 to 3.55)	3.44 (3.10 to 3.77)*
Midwest	617	2216	259.16	1.48 (1.36 to 1.60)	3.68 (3.53 to 3.84)	3.62 (3.44 to 3.80)*
South	838	3498	317.42	1.31 (1.22 to 1.40)	3.15 (3.05 to 3.26)	3.52 (3.37 to 3.68)*
West	546	2250	312.09	1.51 (1.39 to 1.64)	3.43 (3.29 to 3.57)	3.08 (2.89 to 3.26)*
Race/ethnicity						
Hispanic	164	1170	613.41	1.69 (1.42 to 1.97)	3.47 (3.27 to 3.68)	2.80 (2.53 to 3.07)*
NH Black	187	1026	448.66	1.22 (1.04 to 1.40)	3.44 (3.22 to 3.66)	4.03 (3.76 to 4.30)*
NH White	2092	6804	225.24	1.43 (1.37 to 1.50)	3.30 (3.22 to 3.38)	3.43 (3.29 to 3.56)*
NH Other	106	730	588.68	1.97 (1.57 to 2.37)	3.44 (3.19 to 3.70)	1.63 (1.20 to 2.06)*

Deaths were restricted to adults aged 25 years or older with intrahepatic cholangiocarcinoma (ICD-10 C22.1) as the underlying cause of death. AAMRs were age-adjusted to the 2000 U.S. standard population and expressed per 100,000 population. Percent change was calculated from endpoint death counts, and AAPCs were estimated using Joinpoint regression. * indicates a statistically significant AAPC. Subgroup analyses were exploratory and not adjusted for multiple comparisons. Race/ethnicity categories may not sum to the total because of missing or unknown data. AAMR, age-adjusted mortality rate; CI, confidence interval; AAPC, average annual percent change; NH, non-Hispanic.

### 3.2 Differences by sex

ICC mortality increased significantly in both men and women (**Table 1** and **Figure 1**). In 2024, 4,912 deaths occurred among men and 4,840 among women. The AAMR was higher in men than in women (3.70 per 100,000 [95% CI, 3.59–3.80] vs 3.11 per 100,000 [95% CI, 3.02–3.20]). Compared with 1999, the number of deaths increased by 306.62% among men and 260.12% among women. The AAMR increased more rapidly among women (AAPC, 3.56%; 95% CI, 3.39%–3.73%) than among men (AAPC, 3.15%; 95% CI, 3.00%–3.30%).

### 3.3 Differences by age

**Figure 2:**
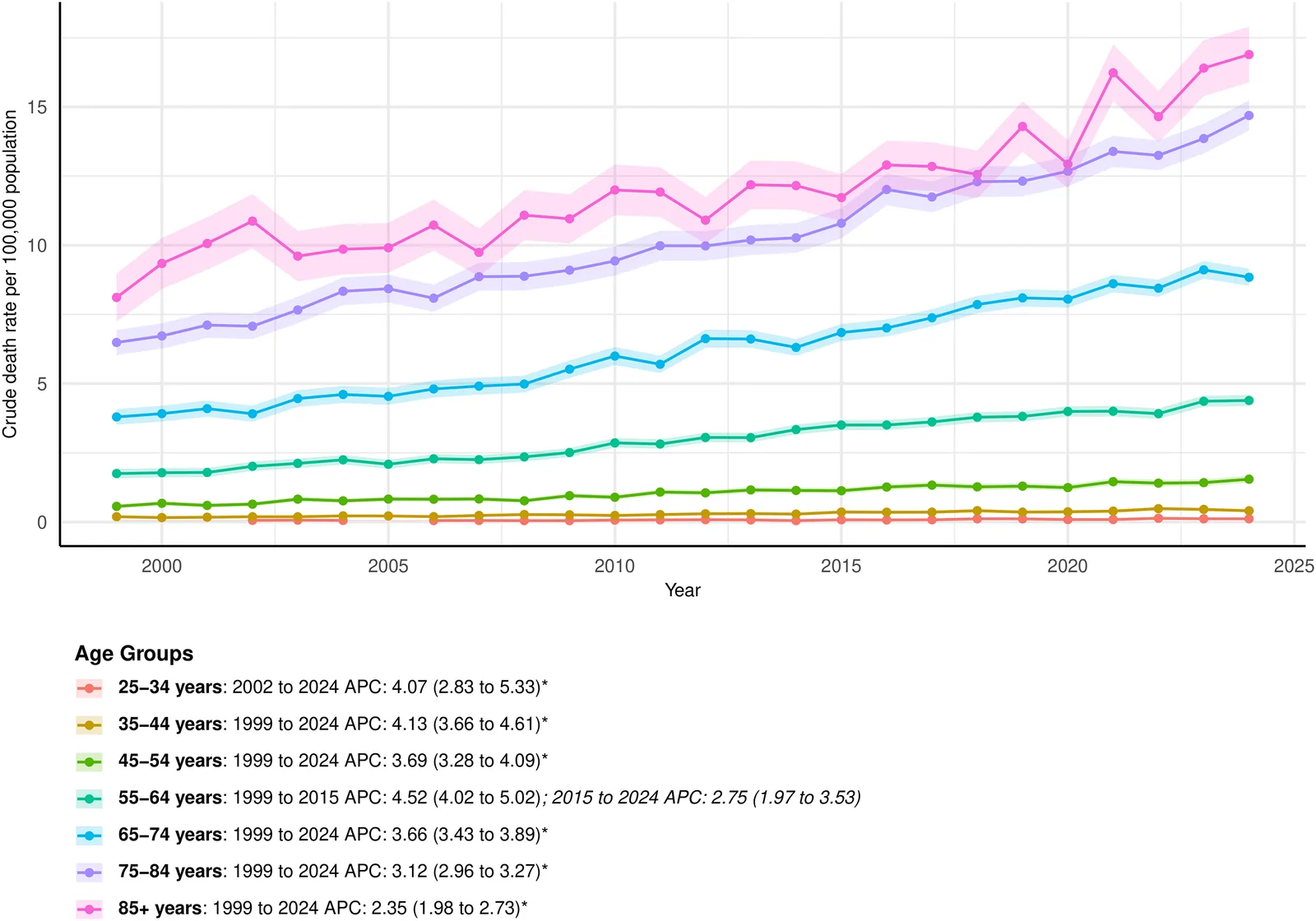
Trends in crude mortality rates for intrahepatic cholangiocarcinoma by age group in the United States, 1999–2024. Age-stratified estimates are age-specific crude mortality rates per 100,000 population. Shaded areas represent 95% CIs. Asterisks indicate statistically significant APCs. APC, annual percent change.

Age-specific crude mortality rates increased with age (**Table 2**; **Figure 2**). In 2024, adults aged 85 years or older had the highest crude mortality rate (16.89 per 100,000; 95% CI, 15.89–17.90), followed by those aged 75–84 years (14.69 per 100,000; 95% CI, 14.15–15.23) and 65–74 years (8.84 per 100,000; 95% CI, 8.53–9.15). Although absolute mortality was lower in younger adults, relative increases were larger. The highest AAPC was observed among adults aged 35–44 years (4.13%; 95% CI, 3.66%–4.61%), followed by those aged 25–34 years (4.07%; 95% CI, 2.83%–5.33%), 55–64 years (3.88%; 95% CI, 3.48%–4.28%), and 45–54 years (3.69%; 95% CI, 3.28%–4.09%). Age-specific crude mortality rates increased significantly in every age group.

**Table 2:** Deaths, age-specific crude mortality rates, and temporal trends of intrahepatic cholangiocarcinoma deaths by age group in the United States, 1999–2024.

Age group	Deaths			Crude mortality rate		
	1999	2024	Percent change (%)	1999 (95% CI)	2024 (95% CI)	AAPC (95% CI)
25-34 years	16	53	231.25	NA (0.02 to 0.06)	0.11 (0.09 to 0.15)	4.07 (2.83 to 5.33)*
35-44 years	85	184	116.47	0.19 (0.15 to 0.23)	0.40 (0.35 to 0.46)	4.13 (3.66 to 4.61)*
45-54 years	206	630	205.83	0.56 (0.49 to 0.64)	1.54 (1.42 to 1.67)	3.69 (3.28 to 4.09)*
55-64 years	416	1829	339.66	1.75 (1.58 to 1.92)	4.39 (4.19 to 4.59)	3.88 (3.48 to 4.28)*
65-74 years	699	3134	348.35	3.80 (3.51 to 4.08)	8.84 (8.53 to 9.15)	3.66 (3.43 to 3.89)*
75-84 years	793	2835	257.50	6.49 (6.04 to 6.94)	14.69 (14.15 to 15.23)	3.12 (2.96 to 3.27)*
85+ years	337	1087	222.55	8.11 (7.25 to 8.98)	16.89 (15.89 to 17.90)	2.35 (1.98 to 2.73)*

Deaths were restricted to adults aged 25 years or older with intrahepatic cholangiocarcinoma (ICD-10 C22.1) as the underlying cause of death. Age-specific crude mortality rates are expressed per 100,000 population. Percent change was calculated from endpoint death counts. AAPCs were estimated using Joinpoint regression. * indicates a statistically significant AAPC. Age-group analyses were exploratory and were not adjusted for multiple comparisons. CI, confidence interval; AAPC, average annual percent change.

### 3.4 Differences by race and ethnicity

**Figure 3:**
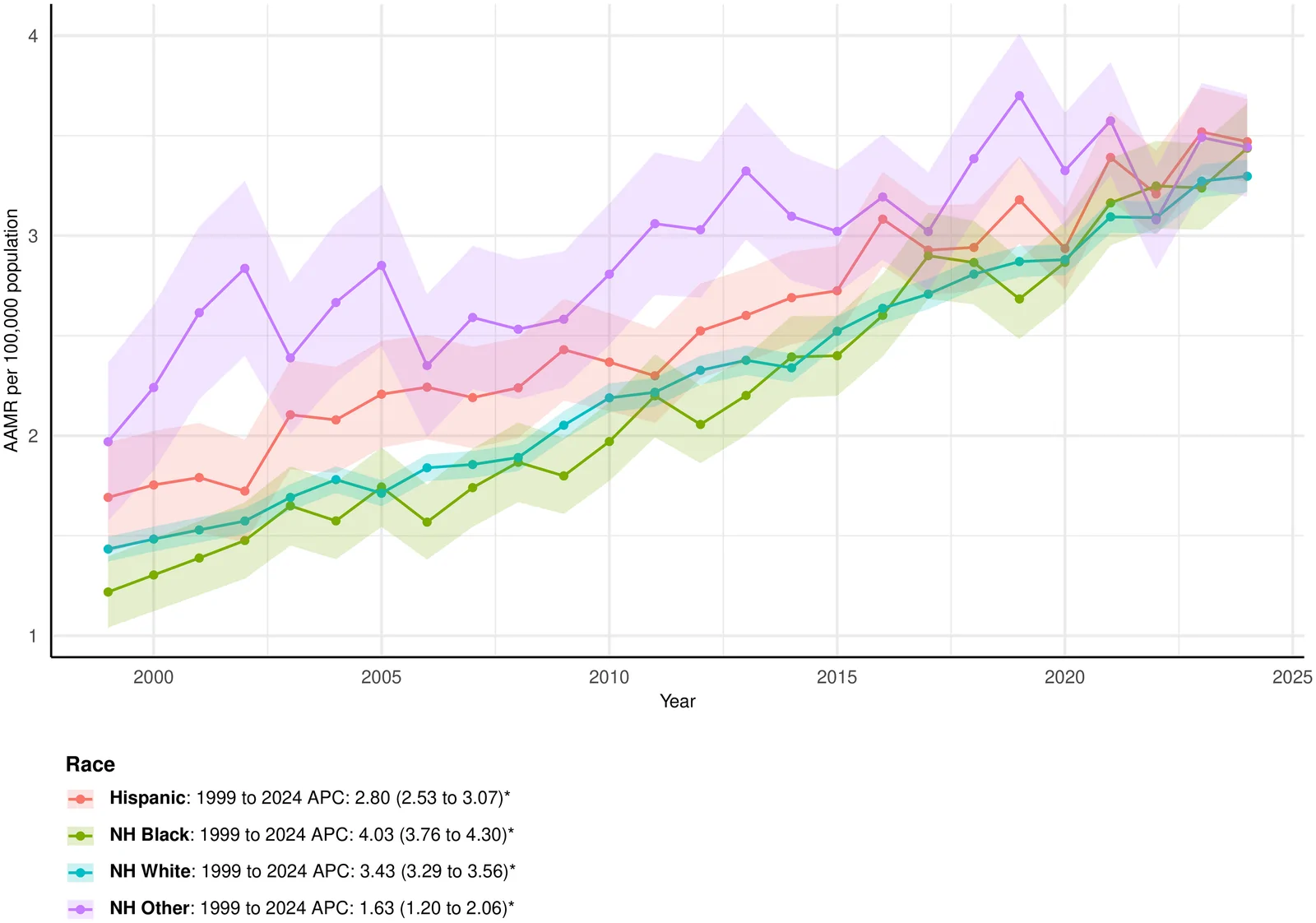
Trends in age-adjusted mortality rates for intrahepatic cholangiocarcinoma by race and ethnicity in the United States, 1999–2024. AAMRs were standardized to the 2000 US standard population and are reported per 100,000 population. Shaded areas represent 95% CIs. Asterisks indicate statistically significant APCs. AAMR, age-adjusted mortality rate; APC, annual percent change; NH, non-Hispanic.

AAMRs increased significantly across all racial and ethnic groups (**Table 1**; **Figure 3**). In 2024, Hispanic individuals had the highest AAMR (3.47 per 100,000; 95% CI, 3.27–3.68). The AAMRs among non-Hispanic Black individuals and non-Hispanic individuals of other races were both 3.44 per 100,000 (95% CIs, 3.22–3.66 and 3.19–3.70, respectively), compared with 3.30 per 100,000 (95% CI, 3.22–3.38) among non-Hispanic White individuals. Non-Hispanic White individuals accounted for the largest cumulative number of deaths (106,524). The largest AAPC occurred among non-Hispanic Black individuals (4.03%; 95% CI, 3.76%–4.30%), followed by non-Hispanic White individuals (3.43%; 95% CI, 3.29%–3.56%), Hispanic individuals (2.80%; 95% CI, 2.53%–3.07%), and non-Hispanic individuals of other races (1.63%; 95% CI, 1.20%–2.06%). Among Hispanic individuals, annual deaths increased from 164 in 1999 to 1,170 in 2024, a 613.41% increase.

### 3.5 Differences by US Census region

**Figure 4:**
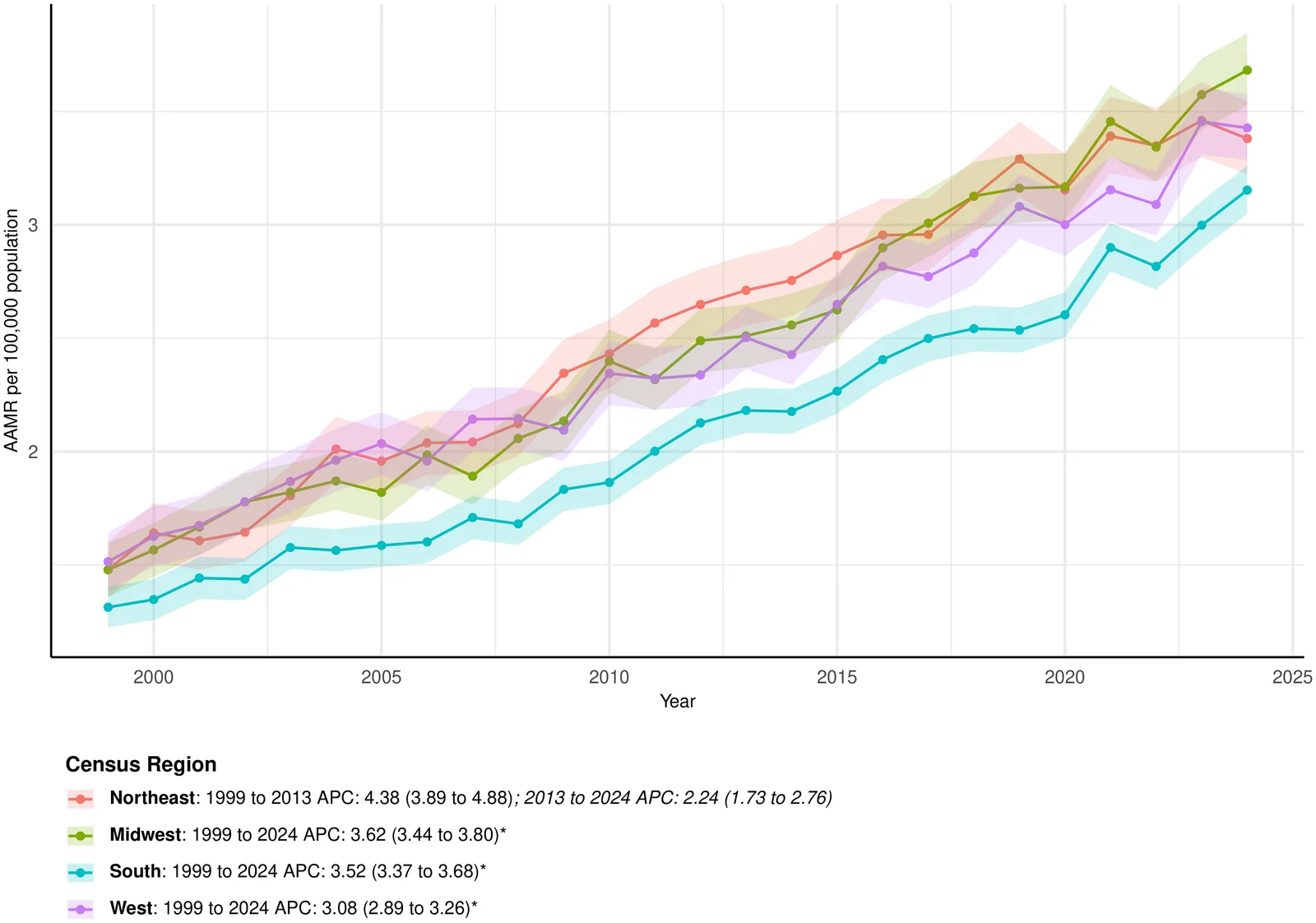
Trends in age-adjusted mortality rates for intrahepatic cholangiocarcinoma by US Census region, 1999–2024. AAMRs were standardized to the 2000 US standard population and are reported per 100,000 population. Shaded areas represent 95% CIs. Asterisks indicate statistically significant APCs. AAMR, age-adjusted mortality rate; APC, annual percent change.

AAMRs increased significantly in all four Census regions (**Table 1**; **Figure 4**). The South had the largest cumulative number of deaths (47,829) and the largest number in 2024 (3,498). The Midwest had the highest AAMR in 2024 (3.68 per 100,000; 95% CI, 3.53–3.84), followed by the West (3.43 per 100,000; 95% CI, 3.29–3.57), Northeast (3.38 per 100,000; 95% CI, 3.22–3.55), and South (3.15 per 100,000; 95% CI, 3.05–3.26). The Midwest also had the largest AAPC (3.62%; 95% CI, 3.44%–3.80%), followed by the South (3.52%; 95% CI, 3.37%–3.68%), Northeast (3.44%; 95% CI, 3.10%–3.77%), and West (3.08%; 95% CI, 2.89%–3.26%).

### 3.6 State-level differences

**Figure 5:**
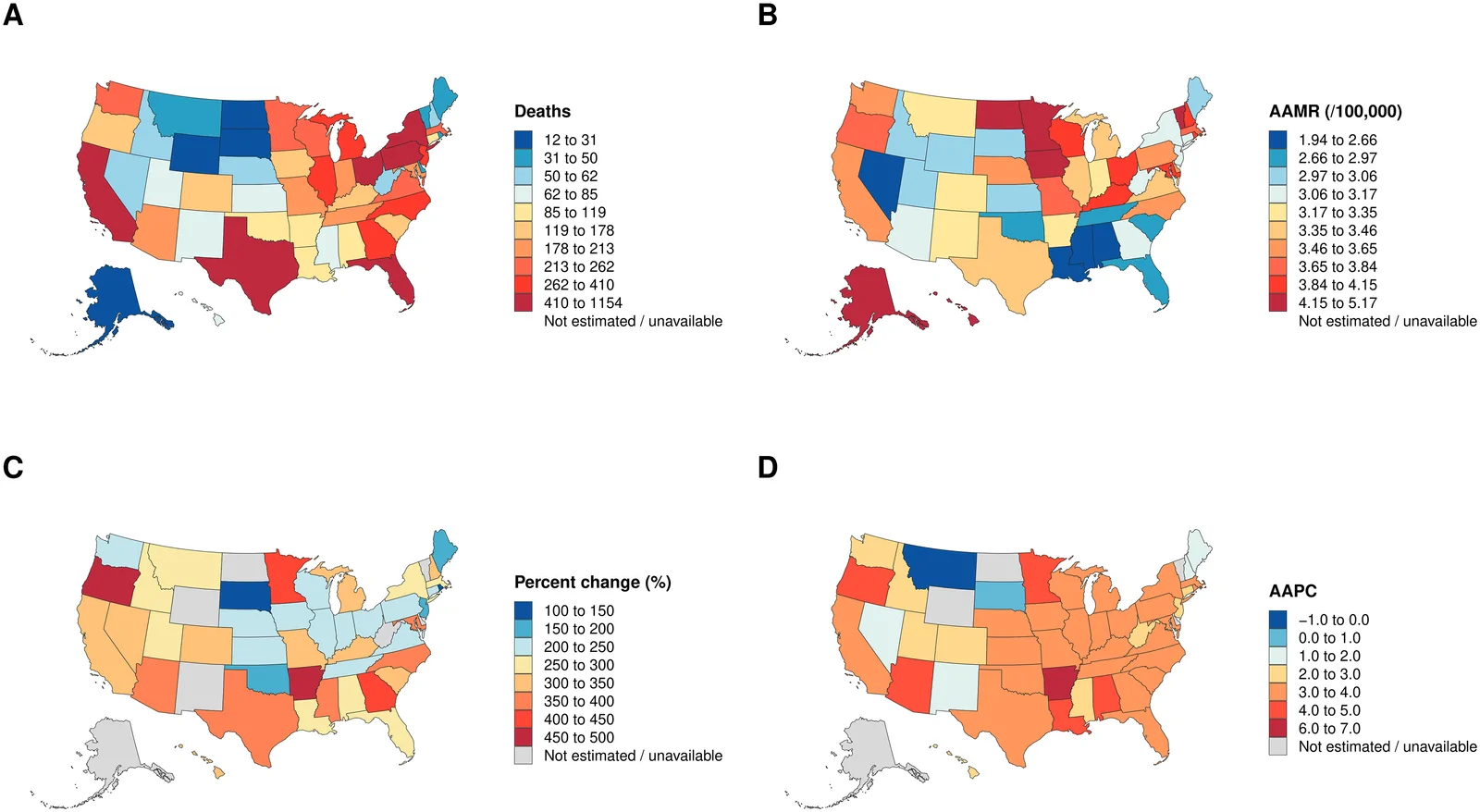
Geographic distribution of the state-level burden of intrahepatic cholangiocarcinoma mortality in the United States. Panel **(A)** shows the number of deaths in 2024; panel **(B)** the AAMR in 2024; panel **(C)** the percentage change in the number of deaths from 1999 through 2024; and panel **(D)** the AAPC from 1999 through 2024. AAMRs were standardized to the 2000 US standard population and are reported per 100,000 population. Not estimated or unavailable indicates suppressed or missing data. AAMR, age-adjusted mortality rate; AAPC, average annual percent change.

State-level analyses showed geographic variation in ICC mortality in 2024 (**Supplementary Table 1**; **Figure 5**). California, Texas, Florida, New York, and Pennsylvania had the largest numbers of deaths (1,154, 732, 684, 568, and 439, respectively). Numerically high AAMRs were observed in Alaska (5.17 per 100,000; 95% CI, 3.32–7.84), Minnesota (4.59 per 100,000; 95% CI, 4.01–5.25), Vermont (4.58 per 100,000; 95% CI, 3.06–6.81), Iowa (4.42 per 100,000; 95% CI, 3.64–5.34), and Hawaii (4.35 per 100,000; 95% CI, 3.34–5.66). Because CIs were wide in several small-population states, these estimates should not be interpreted as a definitive rank ordering. Numerically large AAPCs were observed in Arkansas (6.26%; 95% CI, 4.95%–7.58%), Minnesota (4.84%; 95% CI, 2.35%–7.39%), Arizona (4.58%; 95% CI, 3.74%–5.42%), Louisiana (4.18%; 95% CI, 3.26%–5.11%), and Alabama (4.05%; 95% CI, 3.15%–4.96%). For several states, early-year counts, AAMRs, or relative changes were unavailable because of sparse data or CDC WONDER suppression rules, which further limited precision.

### 3.7 Urban–rural differences

**Figure 6:**
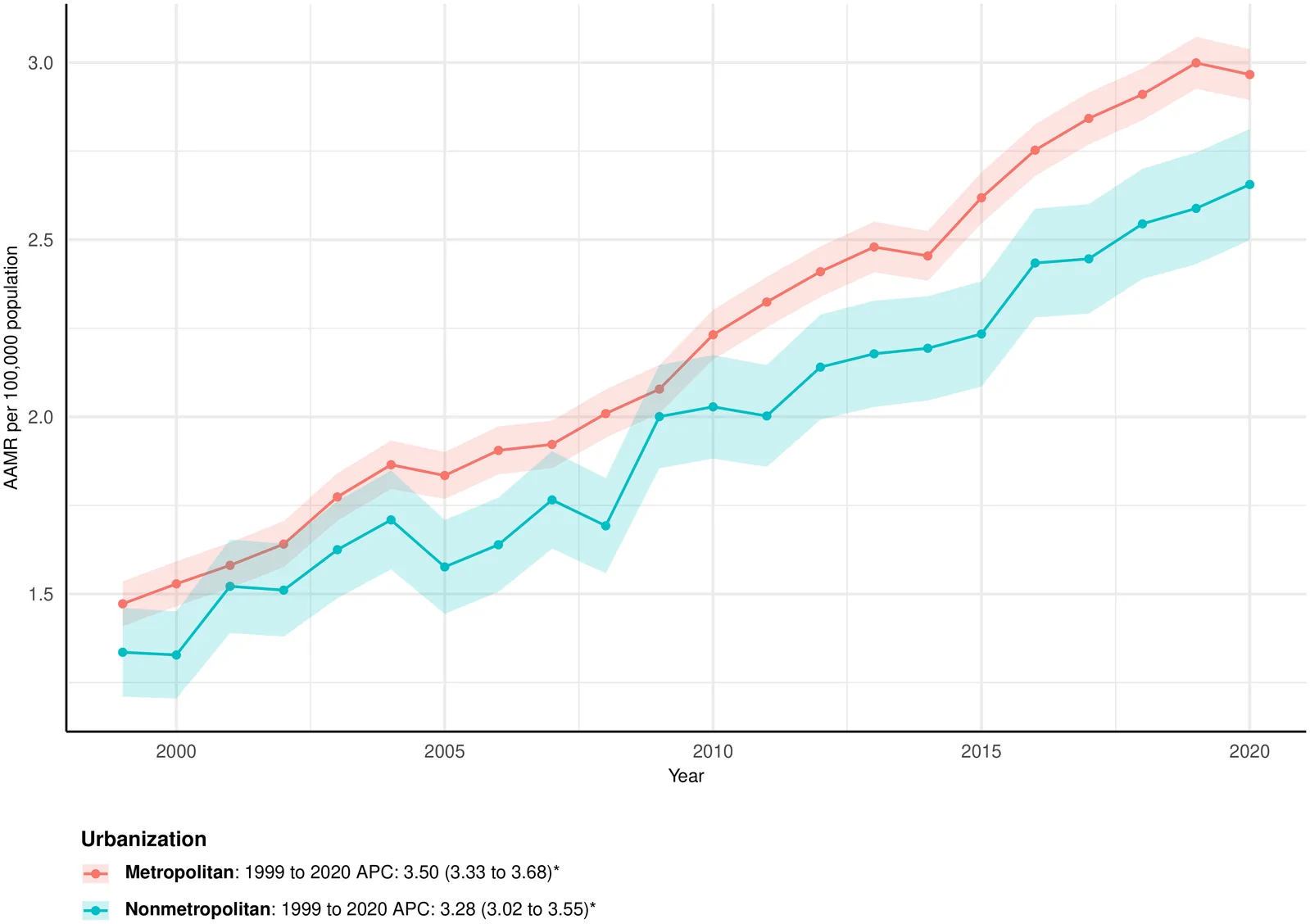
Trends in age-adjusted mortality rates for intrahepatic cholangiocarcinoma in metropolitan and nonmetropolitan areas of the United States, 1999–2020. Urban–rural analyses were restricted to 1999–2020. AAMRs were standardized to the 2000 US standard population and are reported per 100,000 population. Shaded areas represent 95% CIs. Asterisks indicate statistically significant APCs. AAMR, age-adjusted mortality rate; APC, annual percent change.

Urban–rural analyses were restricted to 1999–2020 (**Table 3**; **Figure 6**). In metropolitan areas, annual deaths increased from 2,110 to 6,778 (221.23%), and the AAMR increased from 1.47 per 100,000 (95% CI, 1.41–1.54) to 2.97 per 100,000 (95% CI, 2.89–3.04), corresponding to an AAPC of 3.50% (95% CI, 3.33%–3.68%). In nonmetropolitan areas, annual deaths increased from 442 to 1,177 (166.29%), and the AAMR increased from 1.34 per 100,000 (95% CI, 1.21–1.46) to 2.66 per 100,000 (95% CI, 2.50–2.81), with an AAPC of 3.28% (95% CI, 3.02%–3.55%). Thus, AAMRs increased significantly in both settings, with modestly higher AAMRs and a slightly larger AAPC in metropolitan areas.

**Table 3:** Urbanization-stratified deaths, AAMRs, and temporal trends of intrahepatic cholangiocarcinoma deaths in the United States, 1999–2020.

Characteristic	Deaths			AAMR		
	1999	2020	Percent change (%)	1999 (95% CI)	2020 (95% CI)	AAPC (95% CI)
Urbanization						
Metropolitan	2110	6778	221.23	1.47 (1.41 to 1.54)	2.97 (2.89 to 3.04)	3.50 (3.33 to 3.68)*
Nonmetropolitan	442	1177	166.29	1.34 (1.21 to 1.46)	2.66 (2.50 to 2.81)	3.28 (3.02 to 3.55)*

Deaths were restricted to adults aged 25 years or older with intrahepatic cholangiocarcinoma (ICD-10 C22.1) as the underlying cause of death. Urbanization-stratified analyses were restricted to 1999-2020 because urbanization-level data for 2021-2024 were not available in the extracted dataset. AAMRs were age-adjusted to the 2000 U.S. standard population and are expressed per 100,000 population. Percent change was calculated from endpoint death counts. AAPCs were estimated using Joinpoint regression. * indicates a statistically significant AAPC. AAMR, age-adjusted mortality rate; CI, confidence interval; AAPC, average annual percent change.

## 4 Discussion

In this national analysis spanning 26 years, ICC-coded mortality among US adults aged 25 years or older increased substantially, with the AAMR rising from 1.44 to 3.35 per 100,000 population. The increase was observed across all major demographic and geographic strata but was not uniform. Men had a higher AAMR in 2024, while women had a larger long-term AAPC. Older adults bore the greatest absolute mortality burden, while younger and middle-aged adults had some of the largest relative increases. Hispanic individuals had the highest AAMR in 2024, and non-Hispanic Black individuals had the largest AAPC. Geographically, the Midwest had the highest AAMR and largest AAPC, while the South accounted for the greatest number of deaths. These findings extend previous US analyses through 2024 and distinguish absolute mortality burden from relative rates of increase.

The concurrent rise in annual deaths and the AAMR shows that population growth and aging alone do not account for the observed mortality pattern. Mortality data cannot determine whether the increase reflects rising incidence, changes in survival, shifts in competing causes of death, or a combination of these factors. US registry studies have reported increasing ICC incidence, including among younger adults (2, 3, 11, 21), but those findings cannot be directly linked to the death-certificate trends analyzed here. Improved imaging, pathologic classification, and death-certificate coding may also have increased ascertainment or altered classification over time. Accordingly, the present results describe temporal changes in ICC-coded mortality and do not quantify a true epidemiologic increase in ICC or the contribution of any proposed driver.

Men had a higher AAMR than women throughout the study period, consistent with prior population-based analyses (1). Differences in chronic liver disease, primary sclerosing cholangitis, occupational exposures, and tumor biology have been proposed, including sex variation in TP53 alterations and asbestos-related exposure patterns (1). These factors were not measured in CDC WONDER and cannot explain the observed association. The larger AAPC among women warrants investigation of changing metabolic profiles, diagnostic patterns, and treatment access by sex.

Age-specific findings distinguish absolute burden from relative change. Adults aged 75 years or older had the highest crude mortality rates, whereas adults aged 25–44 years had lower rates but some of the largest AAPCs. Potential contributors to increasing ICC burden include obesity, diabetes, metabolic dysfunction-associated steatotic liver disease, primary sclerosing cholangitis, cirrhosis, and chronic hepatitis B or C virus infection (1, 8, 15, 16). Earlier onset or longer duration of metabolic and chronic liver disease could be relevant to younger-adult trends, and registry studies have reported increasing early-onset ICC incidence (11, 21). These mechanisms remain hypotheses because CDC WONDER contains no individual risk-factor, incidence, or survival data, and relative changes in younger groups may be amplified by low baseline rates and small counts. The findings support further etiologic research and clinical awareness, not population-wide screening of young adults.

Racial and ethnic patterns also differed by burden measure. Hispanic individuals had the highest AAMR in 2024, whereas non-Hispanic Black individuals had the largest AAPC. Prior US studies have documented racial and ethnic disparities in liver disease and cancer mortality (17–20). Metabolic disease, chronic viral hepatitis, cirrhosis, insurance coverage, continuity of care, referral pathways, and access to specialized hepatobiliary oncology are plausible contributors, but none were measured in this study. The broad categories available in CDC WONDER obscure within-group heterogeneity, and the transition from bridged-race to single-race data may affect continuity across 2020–2021. These population-level associations remain subject to residual confounding by unmeasured clinical, socioeconomic, and healthcare factors and should not be interpreted as evidence of biological susceptibility.

Geographic patterns also differed by burden measure. The South had the largest number of deaths, while the Midwest had the highest AAMR and largest AAPC. At the state level, several small-population states had numerically high AAMRs or AAPCs, but wide CIs, suppressed early-year values, and unstable estimates preclude firm rank ordering. Populous states such as California, Texas, and Florida contributed the greatest numbers of deaths and may have the largest implications for service capacity (10). Regional variation may be associated with differences in risk-factor prevalence, diagnostic intensity, referral patterns, access to hepatobiliary centers, and end-of-life care (22, 23). The urban–rural comparison ended in 2020, and the ecological design cannot attribute geographic differences to any single mechanism.

These descriptive findings may inform hypothesis generation and resource planning. No population-wide screening program for ICC has been established. Established risk factors support evidence-based surveillance of clinically defined high-risk groups, and regions with a large absolute burden may require adequate diagnostic, multidisciplinary, surgical, and systemic-treatment capacity (24–26). This analysis did not evaluate screening performance, treatment access, treatment effectiveness, or prognosis and cannot establish that any specific intervention would reduce mortality.

This study has several strengths. It used a nationally representative mortality database, covered 26 years, incorporated data through 2024, and evaluated multiple demographic and geographic dimensions using standardized mortality measures and Joinpoint regression. The study also separately considered absolute death counts, mortality rates, and rates of temporal change, which provide complementary information for public health planning.

Several limitations should be acknowledged. First, CDC WONDER relies on death certificates and is subject to errors in the assigned underlying cause of death, changes in diagnostic practice, and coding variation. ICD-10 code C22.1 does not provide histologic or anatomic confirmation; miscoding of metastatic adenocarcinoma, perihilar or extrahepatic cholangiocarcinoma, or other primary liver and biliary tract cancers could bias levels and temporal trends in either direction. Second, the database lacks incidence, survival, tumor stage, histologic subtype, molecular alterations, treatment, comorbidities, socioeconomic status, insurance, healthcare utilization, and individual risk-factor exposures. The analysis therefore cannot distinguish whether increasing mortality reflects incidence, survival, competing mortality, diagnostic ascertainment, or coding changes, and residual confounding cannot be controlled. Third, bridged-race and single-race classifications are not fully equivalent, which may affect longitudinal race and ethnicity comparisons. Fourth, urban–rural analyses ended in 2020 and do not represent 2021–2024. Fifth, subgroup and state-level analyses were exploratory and were not adjusted for multiple comparisons. Sixth, age-specific analyses used crude rates, and state estimates were sometimes suppressed or imprecise because of small counts. Finally, this ecological analysis cannot determine whether group-level associations apply to individual patients.

## 5 Conclusions

Mortality coded to ICC increased markedly in the United States from 1999 through 2024, with the AAMR rising from 1.44 to 3.35 per 100,000 population. Absolute burden and relative change did not fully overlap: older adults and the South had the greatest absolute burden, while younger adults and the Midwest showed faster relative increases; sex and race and ethnicity patterns also differed by measure. These descriptive trends do not establish changes in ICC incidence or survival, causal mechanisms, individual risk, prognosis, or the effectiveness of clinical interventions. Future studies linking mortality records with cancer registry, clinical, treatment, and social determinants data are needed to identify modifiable contributors and evaluate strategies for equitable care.

## Data Availability

The original contributions presented in the study are included in the article/**Supplementary Material**. Further inquiries can be directed to the corresponding author.
