# Supplementary material for: Trends and disparities in intrahepatic cholangiocarcinoma mortality in the United States, 1999–2024: a CDC WONDER analysis
Source: Front Oncol. 2026 Aug 14;16:1910061. doi: 10.3389/fonc.2026.1910061 (PMC13522743; doi:10.3389/fonc.2026.1910061)
Supplement: Supplementary file 1 [file Table1.docx]

**Supplementary Table 1. State-level deaths, age-adjusted mortality rates, and temporal trends of intrahepatic cholangiocarcinoma deaths in the United States, 1999–2024.**

| **State** | **Deaths** | | |  | **AAMR** | | |
| --- | --- | --- | --- | --- | --- | --- | --- |
|  | **1999** | **2024** | **Percent Change (%)** |  | **1999 (95% CI)** | **2024 (95% CI)** | **AAPC (95% CI)** |
| Alabama | 30 | 112 | 273.33 |  | 1.03 (0.69 to 1.47) | 2.46 (2.01 to 2.99) | 4.05 (3.15 to 4.96)* |
| Alaska | - | 26 | - |  | Suppressed | 5.17 (3.32 to 7.84) | -1.64 (-23.74 to 26.85) |
| Arizona | 44 | 202 | 359.09 |  | 1.39 (1.01 to 1.87) | 3.11 (2.68 to 3.59) | 4.58 (3.74 to 5.42)* |
| Arkansas | 15 | 85 | 466.67 |  | Suppressed | 3.24 (2.58 to 4.06) | 6.26 (4.95 to 7.58)* |
| California | 285 | 1154 | 304.91 |  | 1.55 (1.37 to 1.73) | 3.65 (3.44 to 3.87) | 3.07 (2.87 to 3.28)* |
| Colorado | 35 | 148 | 322.86 |  | 1.61 (1.12 to 2.24) | 3.18 (2.68 to 3.75) | 2.88 (2.15 to 3.61)* |
| Connecticut | 34 | 105 | 208.82 |  | 1.43 (0.99 to 2.00) | 3.06 (2.50 to 3.75) | 2.75 (1.29 to 4.23)* |
| Delaware | - | 38 | - |  | Suppressed | 3.62 (2.53 to 5.16) | 1.40 (-4.80 to 8.01) |
| District of Columbia | - | 12 | - |  | Suppressed | 2.58 (1.32 to 4.63) | -9.72 (-24.02 to 7.27) |
| Florida | 172 | 684 | 297.67 |  | 1.31 (1.11 to 1.51) | 2.91 (2.69 to 3.15) | 3.03 (2.67 to 3.39)* |
| Georgia | 52 | 267 | 413.46 |  | 1.22 (0.91 to 1.60) | 3.12 (2.75 to 3.53) | 3.75 (3.27 to 4.24)* |
| Hawaii | 16 | 65 | 306.25 |  | Suppressed | 4.35 (3.34 to 5.66) | 2.68 (1.48 to 3.89)* |
| Idaho | 14 | 50 | 257.14 |  | Suppressed | 2.97 (2.20 to 3.98) | 2.37 (-0.38 to 5.19) |
| Illinois | 121 | 367 | 203.31 |  | 1.58 (1.30 to 1.86) | 3.39 (3.05 to 3.77) | 3.37 (2.58 to 4.17)* |
| Indiana | 56 | 186 | 232.14 |  | 1.45 (1.09 to 1.88) | 3.20 (2.75 to 3.72) | 3.28 (2.70 to 3.86)* |
| Iowa | 38 | 119 | 213.16 |  | 1.70 (1.20 to 2.35) | 4.42 (3.64 to 5.34) | 3.74 (2.95 to 4.53)* |
| Kansas | 23 | 71 | 208.70 |  | 1.33 (0.84 to 1.99) | 2.97 (2.31 to 3.80) | 3.13 (2.16 to 4.12)* |
| Kentucky | 39 | 156 | 300.00 |  | 1.51 (1.07 to 2.06) | 3.91 (3.30 to 4.61) | 3.96 (3.34 to 4.58)* |
| Louisiana | 26 | 98 | 276.92 |  | 0.99 (0.65 to 1.45) | 2.54 (2.05 to 3.12) | 4.18 (3.26 to 5.11)* |
| Maine | 17 | 47 | 176.47 |  | Suppressed | 3.02 (2.20 to 4.16) | 1.06 (-0.04 to 2.18) |
| Maryland | 43 | 207 | 381.40 |  | 1.35 (0.98 to 1.82) | 3.84 (3.33 to 4.42) | 3.28 (2.72 to 3.84)* |
| Massachusetts | 66 | 237 | 259.09 |  | 1.53 (1.18 to 1.94) | 3.72 (3.25 to 4.24) | 3.83 (2.60 to 5.07)* |
| Michigan | 73 | 315 | 331.51 |  | 1.18 (0.92 to 1.48) | 3.44 (3.06 to 3.87) | 3.85 (3.34 to 4.36)* |
| Minnesota | 43 | 231 | 437.21 |  | 1.41 (1.02 to 1.90) | 4.59 (4.01 to 5.25) | 4.84 (2.35 to 7.39)* |
| Mississippi | 14 | 63 | 350.00 |  | Suppressed | 2.48 (1.89 to 3.24) | 2.89 (1.34 to 4.47)* |
| Missouri | 47 | 197 | 319.15 |  | 1.26 (0.93 to 1.67) | 3.70 (3.19 to 4.28) | 3.43 (2.80 to 4.07)* |
| Montana | 10 | 36 | 260.00 |  | Suppressed | 3.35 (2.32 to 4.78) | -0.15 (-3.04 to 2.82) |
| Nebraska | 17 | 58 | 241.18 |  | Suppressed | 3.58 (2.71 to 4.68) | 2.88 (0.15 to 5.67)* |
| Nevada | 13 | 55 | 323.08 |  | Suppressed | 1.94 (1.45 to 2.57) | 1.51 (0.07 to 2.96)* |
| New Hampshire | 13 | 55 | 323.08 |  | Suppressed | 3.84 (2.87 to 5.12) | 1.91 (0.63 to 3.21)* |
| New Jersey | 93 | 262 | 181.72 |  | 1.65 (1.33 to 2.02) | 3.17 (2.79 to 3.59) | 2.78 (2.25 to 3.32)* |
| New Mexico | - | 62 | - |  | Suppressed | 3.30 (2.50 to 4.31) | 1.99 (0.89 to 3.10)* |
| New York | 162 | 568 | 250.62 |  | 1.31 (1.11 to 1.52) | 3.17 (2.91 to 3.45) | 3.84 (2.98 to 4.71)* |
| North Carolina | 70 | 329 | 370.00 |  | 1.43 (1.11 to 1.81) | 3.56 (3.18 to 3.98) | 3.45 (2.99 to 3.91)* |
| North Dakota | - | 26 | - |  | Suppressed | 4.15 (2.67 to 6.30) | -0.18 (-4.28 to 4.10) |
| Ohio | 118 | 410 | 247.46 |  | 1.57 (1.28 to 1.85) | 3.87 (3.50 to 4.28) | 3.90 (3.53 to 4.28)* |
| Oklahoma | 31 | 91 | 193.55 |  | 1.34 (0.91 to 1.90) | 2.77 (2.22 to 3.43) | 3.33 (2.30 to 4.37)* |
| Oregon | 25 | 145 | 480.00 |  | 1.11 (0.72 to 1.64) | 3.67 (3.09 to 4.36) | 4.02 (3.10 to 4.96)* |
| Pennsylvania | 141 | 439 | 211.35 |  | 1.50 (1.25 to 1.75) | 3.57 (3.23 to 3.94) | 3.53 (3.14 to 3.92)* |
| Rhode Island | 20 | 44 | 120.00 |  | 2.60 (1.59 to 4.02) | 4.07 (2.95 to 5.59) | 3.04 (1.69 to 4.42)* |
| South Carolina | 34 | 136 | 300.00 |  | 1.36 (0.94 to 1.90) | 2.66 (2.23 to 3.18) | 3.46 (2.79 to 4.15)* |
| South Dakota | 11 | 23 | 109.09 |  | Suppressed | 3.04 (1.89 to 4.75) | 0.02 (-3.93 to 4.13) |
| Tennessee | 53 | 178 | 235.85 |  | 1.47 (1.10 to 1.92) | 2.88 (2.46 to 3.35) | 3.42 (2.72 to 4.13)* |
| Texas | 156 | 732 | 369.23 |  | 1.45 (1.22 to 1.67) | 3.38 (3.13 to 3.64) | 3.44 (3.01 to 3.87)* |
| Utah | 17 | 66 | 288.24 |  | Suppressed | 3.05 (2.35 to 3.92) | 2.35 (1.05 to 3.67)* |
| Vermont | - | 31 | - |  | Suppressed | 4.58 (3.06 to 6.81) | -1.61 (-22.21 to 24.45) |
| Virginia | 79 | 254 | 221.52 |  | 1.89 (1.50 to 2.36) | 3.43 (3.01 to 3.89) | 3.32 (2.81 to 3.84)* |
| Washington | 70 | 226 | 222.86 |  | 2.02 (1.57 to 2.55) | 3.46 (3.02 to 3.96) | 2.73 (2.23 to 3.23)* |
| West Virginia | - | 56 | - |  | Suppressed | 3.10 (2.32 to 4.15) | 2.32 (1.46 to 3.19)* |
| Wisconsin | 65 | 213 | 227.69 |  | 1.83 (1.41 to 2.33) | 4.01 (3.48 to 4.62) | 3.01 (2.49 to 3.52)* |
| Wyoming | - | 15 | - |  | Suppressed | 3.05 (1.66 to 5.29) | -5.43 (-10.77 to 0.22) |

**Note:** Deaths were restricted to adults aged 25 years or older with intrahepatic cholangiocarcinoma (ICD-10 C22.1) as the underlying cause of death. *95% CI for AAPC did not include 0. State-level analyses were exploratory and were not adjusted for multiple comparisons. Percent change was calculated as [(deaths in 2024 − deaths in 1999) / deaths in 1999] × 100. AAMRs were age-adjusted to the 2000 U.S. standard population and are reported per 100,000 population. Suppressed indicates that the death count or mortality rate was suppressed by CDC WONDER because the underlying death count was below the reporting threshold. Not estimable indicates that the AAPC could not be estimated because one or more required annual estimates were unavailable or suppressed. AAMR, age-adjusted mortality rate; CI, confidence interval; AAPC, average annual percent change.
